# Ternary Ti alloys functionalised with antibacterial activity

**DOI:** 10.1038/s41598-020-79192-3

**Published:** 2020-12-17

**Authors:** L. Bolzoni, M. Alqattan, L. Peters, Y. Alshammari, F. Yang

**Affiliations:** The University of Waikato, Hamilton, 3240 New Zealand

**Keywords:** Biomaterials, Biomedical engineering

## Abstract

Prosthesis bacterial infection occurring during surgery is a rising health issue. Pathogenic bacterial infection causes inflammation, interferes with the healing process, inhibits osteogenesis and, eventually, leads to implant failure. These issues can be tackled either by applying coatings or developing multifunctional (i.e. structural and antibacterial) materials. In this work, β eutectoid bearing functionalised Ti alloys were designed and manufactured via the cost-effective press and sinter powder metallurgy route. The systematic analysis of the ternary Ti–xCu–yMn alloys shows that the mechanical properties proportionally increase with the amount of alloying elements added. All the ternary Ti–xCu–yMn alloys have strong antibacterial activity against *Escherichia coli* with respect to the negative control (i.e. pure Ti). Our study demonstrates that ternary Ti–xCu–yMn alloys are promising candidates for structural prostheses functionalised with antibacterial capability.

## Introduction

Population aging is a worldwide known phenomenon carrying the consequence that more pressure is placed on public health services which are struggling to cope with the continuously growing demand. With the increased population age, the number of people requiring the fixation or total replacement of a hard-tissue such as hip and knee is, proportionally, increasing. Metals are still the load-bearing material of choice for structural prostheses because of their excellent mechanical properties and cost^1,2^. Among metallic biomaterials (austenitic stainless steel, cobalt-chrome alloys and precious metals), titanium is generally preferred due to its superior biocompatibility and corrosion resistance combined with low density and elastic modulus much more similar to that of human bones^3,4^. However, metallic implants are prone to prosthesis bacterial infection during surgery. This is an increasing issue with infection incidences been reported (10–50% depending on the type of surgery) and clinical trials showing that bacterial infection is one of the most important causes of implants failure^5^. Functionalisation of metallic prostheses to achieve antibacterial activity, which can either be targeted via applying coatings or developing innovative compositions, would greatly improve the success rate of joint replacement surgeries avoiding or limiting infection-related inflammatory reactions which prevent new bone formation^6^.

Commercially pure Ti and Ti–6Al–4V are the most well-established Ti-based implant materials for dental and orthopaedic applications, respectively where corrosion resistance and mechanical requirements is the main sought-after feature. Neither of these materials have antibacterial activity, CP Ti has too low strength for structural joint replacement prostheses^7^, and both are too stiff. Thus, alternative Ti-based compositions (rather than Ti–6Al–4V^8^) for biomedical applications were proposed (e.g. Ti–13Zr–13Nb) but antibacterial infection is still an issue. Furthermore, Cu- and Mn-bearing binary Ti alloys have attracted interest due to the potential to, respectively, promote antibacterial activity and cellular proliferation of human osteoblasts. For example, Liu et al.^9^ analysed the antibacterial activity of binary Ti-(2–25 wt.%)Cu alloys produced at 850-1080 °C via hot pressing at 5–35 MPa for 30–60 min. The Cu content affects the form in which Cu is found in the microstructure and so determines the antibacterial activity as the latter is dependent on the release of Cu^2+^ ions. Mn is known for its cytotoxicity; however, the study of Fernandes Santos et al.^10^ about the mechanical behaviour and cytotoxicity of low cost beta binary Ti-(8–17 wt.%)Mn alloys proved the biocompatibility of these alloys in a simulated body fluids test finding that the antibacterial property is due to the Ti–Mn alloy's ability to form an oxide layer. However, the authors also reported that too high Mn concentrations should be avoided because of known risk of Mn intoxication as well as embrittlement of the alloy. Modification of conventional compositions has also been attempted by Yamanoglu et al.^11^ who added 1–5 wt.% of Cu to the Ti–5Al–2.5Fe alloy hot pressed at 950 °C during 30 min under an applied pressure of 50 MPa. It was found that the antibacterial activity was enhanced by the addition of Cu and the cytotoxicity level remained within the standard values although the cell count was reduced as the amount of Cu increased. Although binary Ti–Cu and Ti–Mn alloys were studied, no reports can be found in literature where these two elements (Cu and Mn) are jointly added to titanium.

This study is therefore a systematic investigation of the combined addition of Cu and Mn to potentially simultaneously achieve antibacterial and cellular proliferation activities in ternary Ti–xCu–yMn alloys. Specifically, β eutectoid bearing functionalised Ti alloys were fabricated via the standard powder metallurgy route of press and sinter where the composition can easily be changed by mixing different starting powders. Moreover, this technique permits to manufacture near net shape structural prostheses at reduced costs. Apart for their biocompatibility and bioactivities, Cu and Mn were chosen as cheap alloying elements primarily because they are stabilisers of the β-Ti phase, which is needed to achieve a good balance of mechanical properties critical for structural joint replacement prostheses. The design strategy used to create these new alloys has been reported elsewhere in a short letter^12^. Characterisation of these novel ternary Ti–xCu–yMn alloys include microstructure, phase identification, tensile properties, fracture mode, hardness and in vitro antibacterial activity against *Escherichia coli* (*E. coli*).

## Results

### Microstructural analysis and phases’ identification

**Figure 1:**
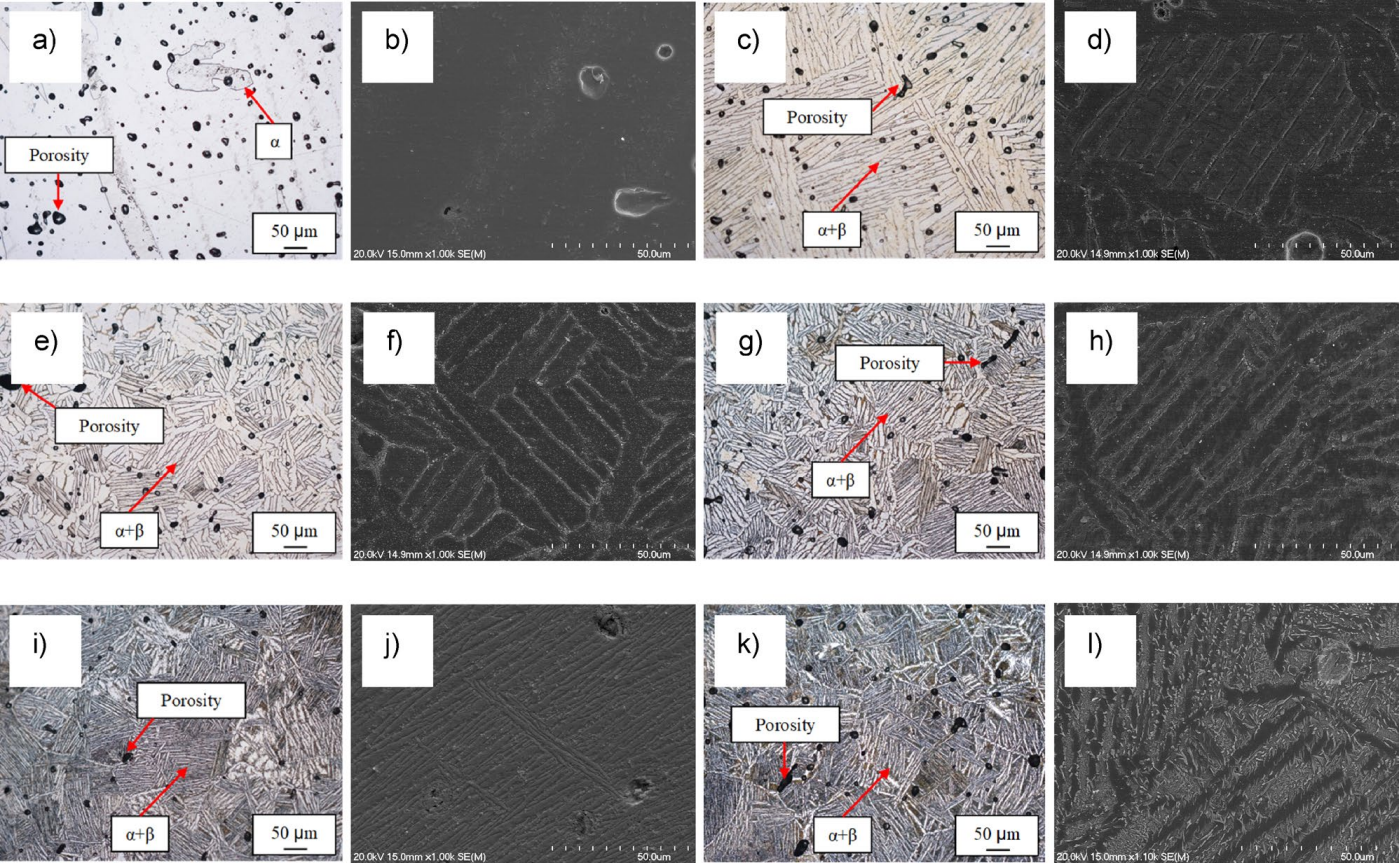
Optical and SEM micrographs, respectively, for: (**a**) and (**b**) CP Ti, (**c**) and (**d**) Ti–0.5Cu–0.5Mn, (**e**) and (**f**) Ti–1Cu–1Mn, (**g**) and (**h**) Ti–2Cu–2Mn, (**i**) and (**j**) Ti–3.5Cu–3.5Mn, and (**k**) and (**l**) Ti–5Cu–5Mn β eutectoid bearing functionalised Ti alloys.

The microstructure of the β eutectoid bearing functionalised Ti alloys was analysed to identify the microfeatures present after sintering. From Fig. 1, it can be seen that regardless of the composition, the sintered Ti-based materials are characterised by the presence of some residual porosity left by the pressureless sintering process. The majority of the pores have a spherical morphology (with a size distribution approximately within 5–50 µm) and they are found at grain and lamellar boundaries.

CP Ti is characterised by equiaxed α-Ti grains and the initial addition of 0.5 wt.% of Cu and Mn switches the microstructure to (coarse) lamellar. The size and interspace of the lamellae is progressively refined by the incremental addition of Cu and Mn but the microstructure of the ternary Ti–xCu–yMn alloys is always composed by α-Ti and α + β lamellae regardless of the chemical composition of the material. It is worth mentioning that a hypoeutectoid substructure was found in the Ti–5Cu–5Mn alloy. Analysis of the microstructure of the β eutectoid bearing functionalised Ti alloys via SEM confirmed the homogeneity of the chemical composition (via EDS) and highlighted the formation of an intermetallic phase for ternary Ti–xCu–yMn alloys with Cu ≥ 2 wt.%. Based on the binary phase diagrams^13^ and the formation of a eutectoid structure, much more clearly noticeable in the Ti–5Cu–5Mn alloys (Fig. 1l), this intermetallic phase is expected to be Ti_2_Cu.

**Figure 2:**
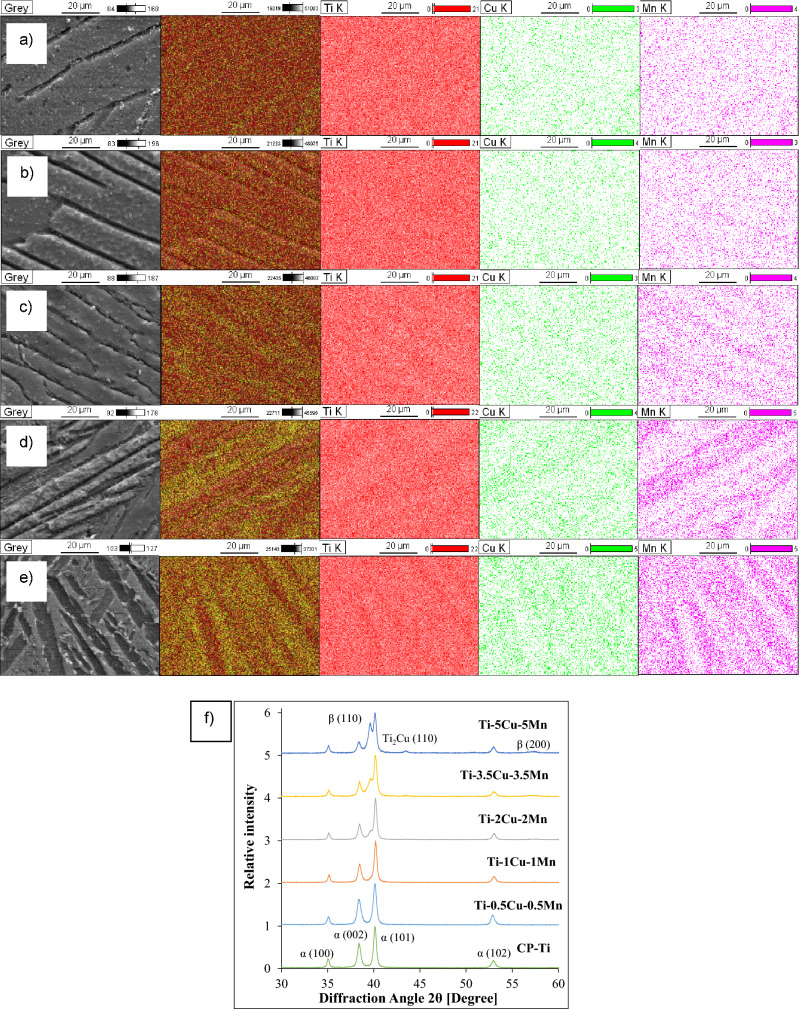
Representative results of the identification of the phases (via elemental mapping) for: (**a**) Ti–0.5Cu–0.5Mn, (**b**) Ti–1Cu–1Mn, (**c**) Ti–2Cu–2Mn, (**d**) Ti–3.5Cu–3.5Mn, and (**e**) Ti–5Cu–5Mn, and (**f**) XRD patterns of the β eutectoid bearing functionalised Ti alloys.

Identification of the phases constituting the β eutectoid bearing functionalised Ti alloys, as well as the homogeneity of the chemistry, and confirmation of the stoichiometry and crystal structure of the intermetallic phase found during microstructural analysis was done by means of elemental mapping and XRD, respectively (Fig. 2). Elemental mapping clearly shows that the sintered β eutectoid bearing functionalised Ti alloys are chemically homogeneous and, as per EDS analysis, the actual alloying element content matches the original addition rate. The alloying elements (i.e. Cu and Mn) are preferentially found in the β-Ti phase. This is in agreement with the respective binary phase diagram which shows that both Cu and Mn have very low solid solubility in the α-Ti phase^13^, which is actually lower for Mn than for Cu. From the XRD spectra (Fig. 2f), there are three main phases found where the relative amount of these phases changes with the amount of alloying elements present in the ternary Ti–xCu–yMn alloys. Specifically, α-Ti is the main phase and β-Ti and Ti_2_Cu are, respectively, detected starting from alloys with composition of Ti–2Cu–2Mn and Ti–3.5Cu–3.5Mn.

### Physical properties

**Figure 3:**
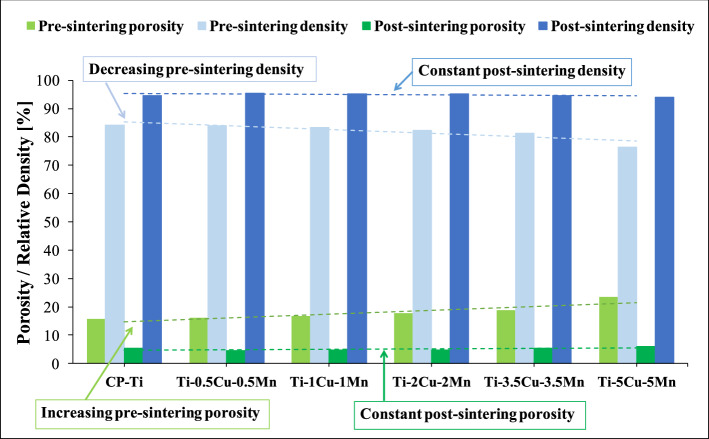
Variation of the porosity and relative density before and after the sintering of CP Ti and of the β eutectoid bearing functionalised Ti alloys.

Ti-based materials manufactured via the conventional powder metallurgy route are, generally, characterised by the presence of porosity both before and after sintering. Figure 3 shows that the porosity left by the compaction of the powder blends increases, and so the density decreases, as the amount of alloying elements increases (i.e. pre-sintering condition). However, the amount of porosity after sintering, and consequently the relative density of the ternary Ti–xCu–yMn alloys is constant (i.e. post-sintering condition). Furthermore, the pre-sintering relative density of Ti–0.5Cu–0.5Mn is the highest (83.9%) compared to the other ternary compositions, Ti–5Cu–5Mn is the lowest (76.5%) and that of CP Ti is highest (84.2%). The difference in terms of density is almost levelled by the sintering process where the highest difference is approximately 1.4%.

### Mechanical behaviour

**Figure 4:**
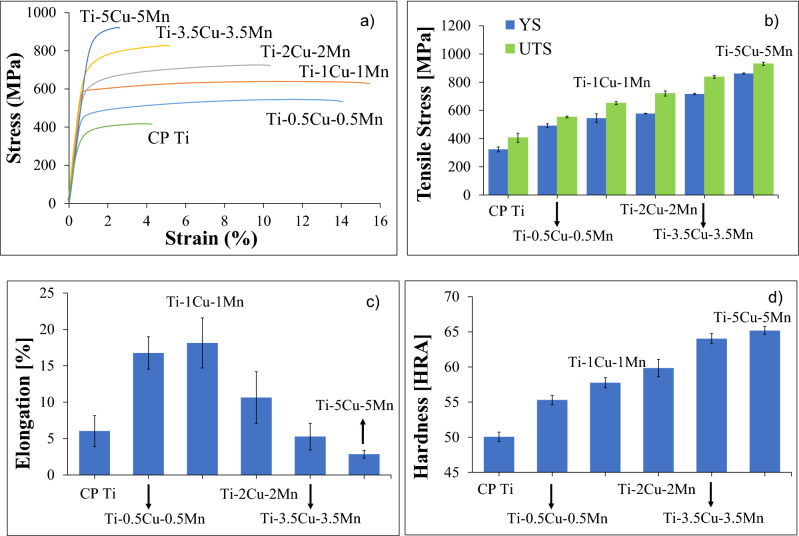
Mechanical behaviour of the β eutectoid bearing functionalised Ti alloys: (**a**) representative engineering stress–strain curves, (**b**) yield and ultimate tensile strength, (**c**) elongation at fracture, and (**d**) Rockwell hardness.

The mechanical behaviour of the β eutectoid bearing functionalised Ti alloys in comparison to CP Ti is presented in Fig. 4 which shows representative examples of the tensile engineering stress–strain curve, average tensile properties (including yield strength (YS), ultimate tensile strength (UTS) and elongation at fracture) and Rockwell hardness. Generally, the resistance to plastic deformation, either tensile or indentation, increases with the amount of alloying elements and it is significantly higher compared to CP Ti but the elongation at fracture initially increases, reaching the highest value for the Ti–1Cu–1Mn alloy, and then decreases. More in detail, CP Ti has the lowest UTS (406 MPa) compared to all sintered Ti alloys, UTS which ranges from 553 to 931 MPa. Correspondingly, the YS shows a consistent increase (from 492 to 862 MPa) as the amount of alloying elements increases compared to CP Ti, which has the lowest value (324 MPa). The sintered Ti–5Cu–5Mn alloy shows the highest YS and UTS among all of the tested specimens (Fig. 4b). Regarding the elongation, the Ti–5Cu–5Mn alloy has the lowest value (2.9%) even when compared to CP Ti (6.0%) as shown in Fig. 4c). The hardness increases linearly (Fig. 4d), ranging from 55 to 65 HRA, as the content of Cu and Mn in the Ti alloys increases and CP Ti has the lowest hardness (50 HRA).

**Figure 5:**
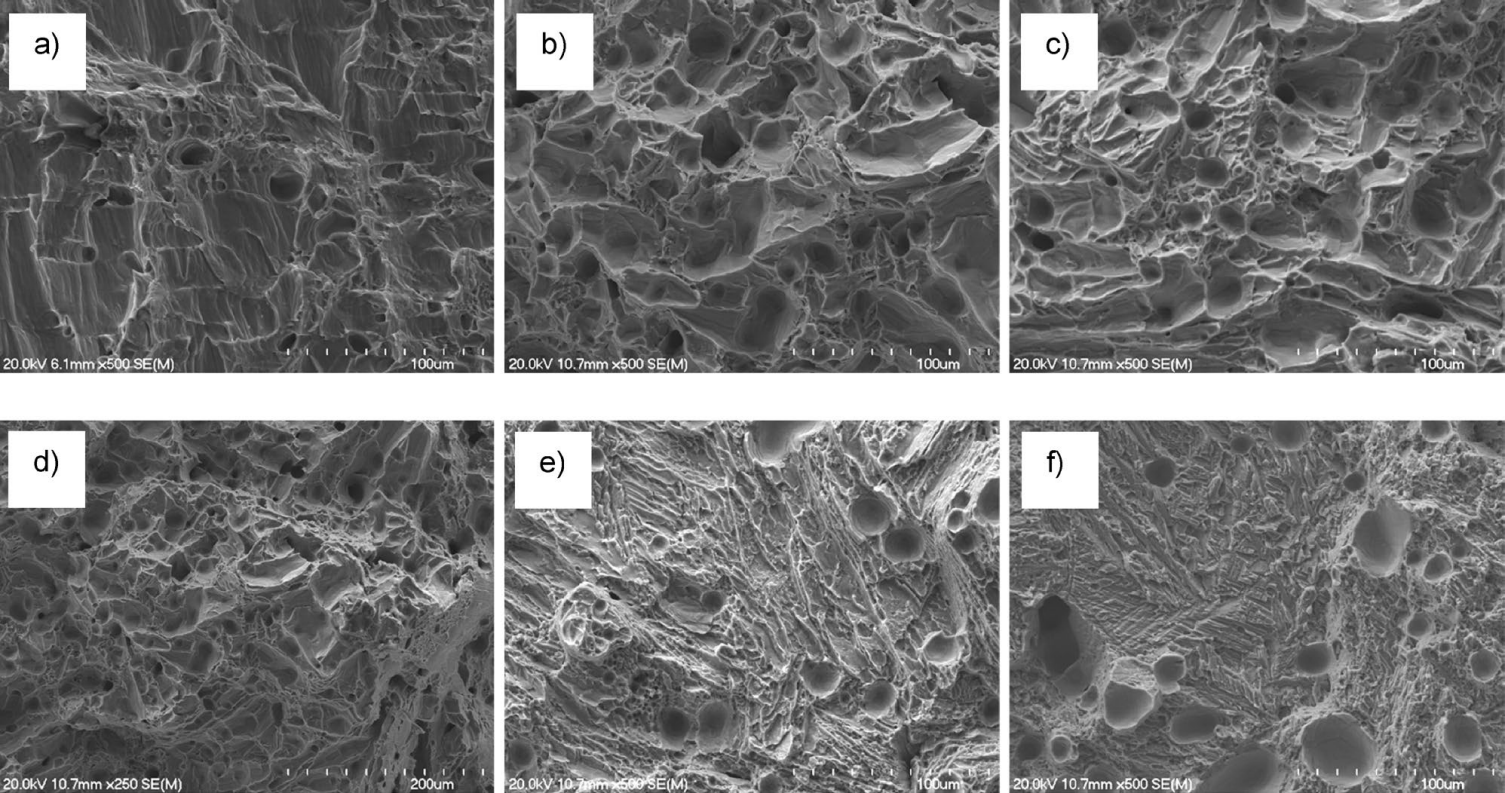
Representative results of the fractographic analysis for: (**a**) CP Ti, (**b**) Ti–0.5Cu–0.5Mn, (**c**) Ti–1Cu–1Mn, (**d**) Ti–2Cu–2Mn, (**e**) Ti–3.5Cu–3.5Mn, and (**f**) Ti–5Cu–5Mn β eutectoid bearing functionalised Ti alloys.

Fractographic analysis permits to better understand the behaviour of a material during deformation. From Fig. 5, it can be seen that the fracture surface of CP Ti, which is completely composed of dimples due to the ductile nature of the fracture, changes due to both the formation of the α + β lamellar structure and the switching of the fracture mode due to the embrittlement of the β eutectoid bearing functionalised Ti alloys. More in detail, transgranular fracture and formation of river-like patterns are present in the Ti–0.5Cu–0.5Mn, Ti–1Cu–1Mn, and Ti–2Cu–2Mn alloys whereas tear-ridges are found in the Ti–3.5Cu–3.5Mn and Ti–5Cu–5Mn alloys as a consequence of the formation of the Ti_2_Cu intermetallic particles and the associated reduction of the ductility and ability to sustain damage prior to failure.

### In vitro* antibacterial activity*

**Figure 6:**
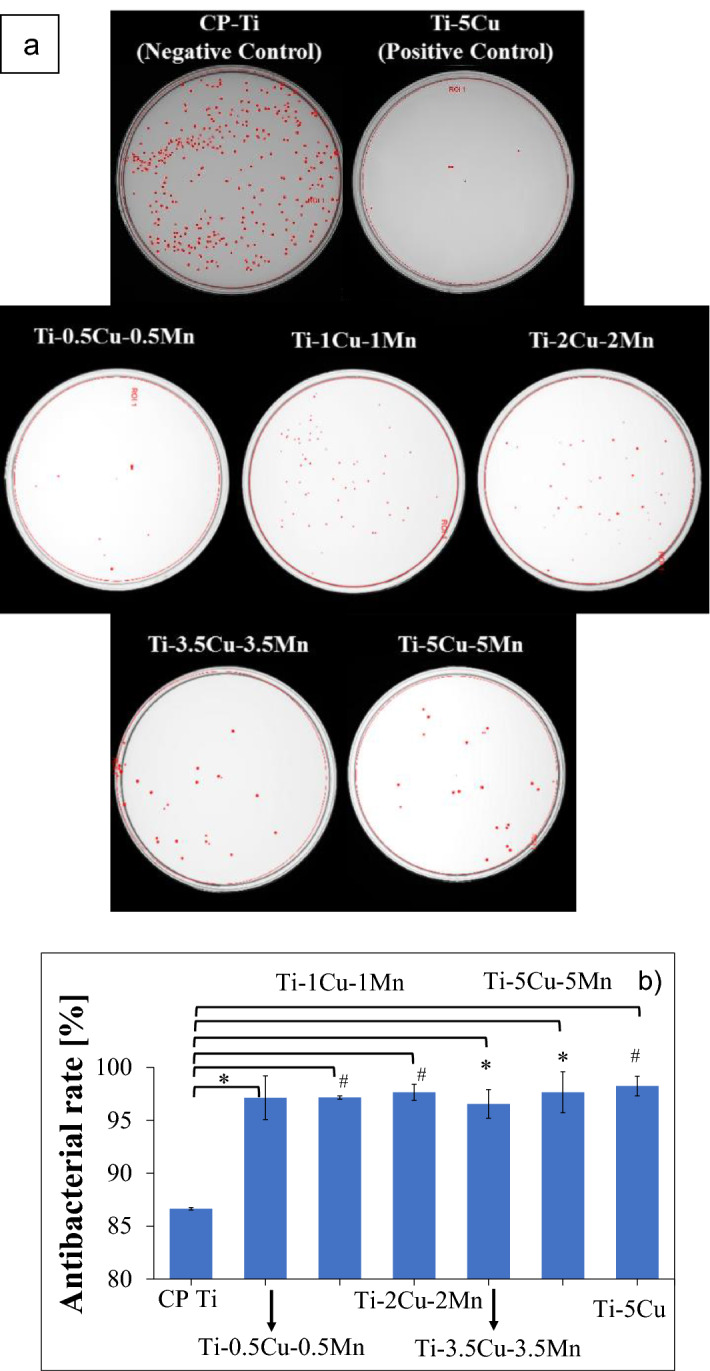
Representative photographs of the *E. coli* bacterial colonies after 24 h incubation for β eutectoid bearing functionalised Ti alloys, (**a**) CP Ti (positive control), and Ti–5Cu (negative control) specimens, and (**b**) respective in vitro antibacterial rate using *E. coli* culture. *Note*: *p < 0.05 and #p < 0.01.

Different combinations of Cu and Mn in Ti–xCu–yMn were tested for antibacterial activity. The result of OD_600_ of an overnight culture averaged 0.80 nm, which is equivalent to 2.84 × 10^11^ CFU/mL (calculated via Eq. 1); therefore, approximately 5.68 × 10^10^ of DH5-α *E. coli* were used for lawn inoculation. Typical photographs of bacterial colonies on the agar plates after overnight (24 h) incubation for ternary Ti–xCu–yMn alloys as well as CP Ti (positive control) and Ti–5Cu (negative control) specimens are shown in Fig. 6 from which it can be seen that the antibacterial performance of the β eutectoid bearing functionalised Ti alloys is very strong and pronounced and comparable to the positive control Ti–5Cu (Fig. 6b). After 24 h incubation, the negative control (CP Ti) has a large number of *E. coli* colonies indicating poor antibacterial properties. From Fig. 6b), sintered CP Ti has antibacterial rate of 86.6% (calculated via Eq. 2), meaning that CP Ti does not feature antibacterial activity as per the National Standard of China (GB/T 4789.2–2010)^14^. The β eutectoid bearing functionalised Ti alloys have antibacterial rate between 96.5% and 97.6%. When comparing sintered Ti–xCu–yMn alloys to sintered Ti–5Cu, the results show a comparable antibacterial rate with Ti–5Cu yielding 98.2%.

## Discussion

Biomaterials are commonly used in biomedicine to repair, replace and regenerate body tissues. Most of the structural metallic biomaterial are intrinsically inert and thus functionalisation can be considered. Among the functions that could be added to a metallic biomaterial, antibacterial activity would be of great importance as an increasing number of implants failure derived by pathogenic bacterial infection during surgery has been reported^5^. For that purpose, the selection of the alloying elements is crucial as the release of alloying metal ions into the human body can provide biofunction like antibacterial activity and enhanced bone regeneration. In this study, Cu and Mn were selected as alloying elements for Ti, where both are essential elements for the human body. For instance, Cu helps in maintaining the daily stability of organs and metabolic processes^15^ and its lacking (as well as that of Mn) affects bone induction and osteoblast proliferation, differentiation and migration^16^. Moreover, Cu^2+^ ions promote angiogenesis in vitro and *in vivo*^17^ and contribute to heal full-thickness skin defects without toxicity^18^. Similarly, Mn is a highly necessary element required for bone and skeletal growth and development as well as an amino acid, lipid, cholesterol, enzymes (superoxide dismutase), and carbohydrate metabolism promoter^19^.

The way a material is produced affects a range of properties, generally explicitly visible in the change of mechanical behaviour and fracture mode, due to the modifications induced at microstructural level, which could also change the functionalisation of metallic biomaterials. Therefore, the selection of the manufacturing process to be used is also crucial. Powder metallurgy is a recognised green technology ideal to make, in few simple steps, near net or net shape products such as total joint prostheses at reduced cost with respect to the conventional metallurgical route. In this study, the samples were pressureless sintered under vacuum and this resulted, as it could expected on the basis of the classical sintering theory, in dense materials with some residual porosity left as microstructural feature of the β eutectoid bearing functionalised Ti alloys (Fig. 1). These pores are mainly spherical in shape and located at grain and lamellar boundaries. This means that, with the sintering parameters used in this study, the materials reached the last stage of sintering^20^ and thus a fully homogeneous chemical composition would be expected, which was actually proved via elemental mapping (Fig. 2). The pores present in the microstructure of the sintered β eutectoid bearing functionalised Ti alloys can have two main effects: affect the mechanical behaviour (as pores act as stress concentration sites) and promote biocompatibility (as bone ingrowth can occur inside the pores).

Figure 3 shows that the addition of an increasing amount of alloying elements to CP Ti decreases the pre-sintering relative density meaning that more pores are left in the pressed material. Generally, a higher pre-sintering porosity results into a higher post-sintering porosity because the efficiency of the sintering process is reduced. However, the post-sintering relative density values shown in Fig. 3 are almost constant (94.9 ± 0.7%) regardless of the chemical composition. This means that a higher amount of Cu and Mn induces a higher densification of the β eutectoid bearing functionalised Ti alloys during sintering. The relative density values shown in Fig. 4 are comparable to those of other Cu- and Mn-bearing Ti alloys produced via powder metallurgy^21,22^.

The incorporation of Cu and Mn into the Ti matrix changes the typical microstructure of CP Ti from equiaxed to lamellar and the processing conditions employed guarantee the achievement of fully homogenised materials (Fig. 2). The amount of α-Ti grains progressively decreases (although the size of the prior β does not seem to be significantly affected) and the size of the α + β lamellae is progressively refined when increasing the amount of alloying elements. The interlamellar spacing (i.e. distance between parallel lamellae within the α-Ti grains) is also significantly refined by the addition of a higher amount of alloying elements. This microstructural changes have a remarkable effect on the mechanical behaviour of the alloy (Fig. 4). However, irrespective of the chemistry of the material, the sintered β eutectoid bearing functionalised Ti alloys are characterised by the typical lamellar microstructure of α + β Ti alloys slow cooled through the β transus temperature^23,24^. This lamellar structure is known for its ability to provide the best compromise between strength, ductility and damage tolerance in biomedical materials such as Ti–6Al–4V^25^.

The incremental addition of the alloying elements (especially Cu) to CP Ti also leads to the formation of a new microstructural features. This was confirmed via XRD analysis to be the Ti_2_Cu intermetallic phase. Specifically, the XRD patterns of Fig. 2f) show that the α-Ti (hexagonal close packed) phase is always the predominant phase as the relative intensity of the strongest α-Ti peak, which is α(101), is always the highest. Although the phase is present in the microstructure, peaks corresponding to the β-Ti (body centred cubic) phase, such as β(110), are detected starting from the Ti–2Cu–2Mn composition and the diffraction intensity of these peaks increases with the amount of alloying elements as both Cu and Mn are β-stabiliser. The absence of the β-Ti phase at a composition of 1 wt.% and lower is due to the fact that the small amount of retained β-Ti phase is below the detection limit of the equipment.

In terms of mechanical behaviour, the ternary Ti–xCu–yMn alloys show both an elastic and plastic deformation region in their stress–strain curves (Fig. 4a). As the different curves overlap in the elastic region, the β eutectoid bearing functionalised Ti alloys have comparable modulus of elasticity. The load at which the alloy starts to deformation plastically is progressively shifted towards higher value as the amount of alloying element increases (Fig. 4a). Consequently, the YS and UTS of the β eutectoid bearing functionalised Ti alloys continuously increase (Fig. 4b). The hardness of the ternary Ti–xCu–yMn alloys also proportionally increases with the amount of alloying elements becoming significantly harder than CP Ti (Fig. 4d).

The increase of the mechanical properties, both tensile strengths and hardness, is due to a combination of different microstructural factors. Specifically, the addition of Cu and Mn promotes the stabilisation at room temperature of the β-Ti which is stronger than the α-Ti phase. The presence of the Cu and Mn atoms within the Ti lattice contributes to the strength via the substitutional solid solution strengthening mechanism. Additionally, the progressive addition of more Cu and Mn leads to the refinement of the microstructural features composing the α + β lamellar structure (Fig. 1) where this refinement is beneficial for hindering the movement of dislocations and, therefore, increase the resistance of the β eutectoid bearing functionalised Ti alloys to plastic deformation. The last strengthening mechanism operating to enhance the strength of the ternary Ti–xCu–yMn alloys is precipitation strengthening due to the formation of an increasing number of fine eutectoid Ti_2_Cu intermetallic particles^26^. This contribution becomes more relevant for the Ti–3.5Cu–3.5Mn and Ti–5Cu–5Mn alloys where the amount of Ti_2_Cu intermetallic phase is high enough to be detected via XRD (Fig. 2f). The presence of residual pores also contributes to the overall mechanical behaviour of the β eutectoid bearing functionalised Ti alloys, actually lowering the strength and hardness with respect to an equivalent fully dense (i.e. without porosity) ternary Ti–xCu–yMn alloy.

As strength and ductility are mutually exclusive properties, the increase in strength is generally accompanied by a decrease of the elongation at fracture. In the case of the β eutectoid bearing functionalised Ti alloys the elongation at fracture starts to decrease for an addition of ≥ 2Cu + 2Mn to CP Ti (Fig. 4c). The initial introduction of a small amount of β-stabilising elements leads to the presence of some β-Ti phase and so to an increase of ductility as the body centred cubic structure of the β-Ti phase adds extra slip systems for accommodating plastic deformation. Nonetheless, as the amount of alloying elements increases and the microstructure is refined, the strength/ductility trade-off becomes predominant and so the ability of the ternary Ti–xCu–yMn alloys to withstand damage before fracturing decreases. The progressive decrease of the elongation at failure starting from the Ti–2Cu–2Mn alloy is also a consequence of the fact that the increase of the amount of alloying elements leads to the formation of the Ti_2_Cu intermetallic phase, whose intrinsic more brittle behaviour in comparison to CP Ti contributes to the embrittlement of the β eutectoid bearing functionalised Ti alloys. Finally, although porosity has an effect on the strength of materials, the residual pores have a significantly higher negative impact on the ductility of the alloys. Specifically, pores act as stress concentration sites and therefore, apart from the amount of pores, their morphology and distribution are also critical as elongated pores have remarkably higher stress concentration factors^27^ and they can provide a preferential pathways for crack growth. From fractographic analysis (Fig. 5), the progressive strengthening and embrittlement of the ternary Ti–xCu–yMn alloys is accompanied by a transition of the fracture mode from pure ductile in CP Ti, whose fracture surface is composed of dimples, to the formation of river-like patterns typical of transgranular fracture along the α-Ti grain boundaries (or prior β grains) of the lamellar microstructure and, eventually, to the creation of tear-ridges associated with the intergranular fracture of the lamellar structures.

The results of the in vitro antibacterial rate using *E. coli* culture (Fig. 6) clearly indicate that ternary Ti–xCu–yMn alloys are characterised by a high antibacterial ability (*R* > 96.5%) which is consistent with the literature (e.g. binary Ti–5Cu alloy). The statistically significant increase in antibacterial activity in comparison to CP Ti is due to the increased amount of alloying elements, Cu in particular, and the formation of a uniform distribution of Ti_2_Cu intermetallic particles. Both effects contribute to a greater metal ions release rate, which improves the antibacterial ability as previously observed for Ti–Cu and the Ti–6Al–4V–5Cu alloys^28,29^. The homogeneous chemistry of the sintered ternary Ti–xCu–yMn alloys confirmed via elemental mapping (Fig. 2), generally found for blended elemental alloys^30^, should ensure a uniform release of metal ions regardless of which surface of the implant will be contact with the human body and potential pathogenic bacteria. The reaction of Cu^2+^ ions with biological proteins such as thiol (-SH) and amino (-NH_2_) present in the nucleic acid reduces the activity of the protein and enzyme (i.e. inhibiting the metabolism), and this has been identified as the killing mechanism of action of Cu^2+^ ions^15^.

β eutectoid bearing functionalised Ti alloys are also expected to have excellent cellular proliferation activity and osteoid formation as proved for other Cu-bearing titanium alloys^31^ as well as the ability to inhibit the formation of biofilms and prevent antibacterial infection associated with dental implants^32^. Moreover, it has been demonstrated that Ti–Cu alloys have good osteoblast adhesion, promote formation of cytoskeleton and cell migration as well as no cytotoxicity to MG63 cell and the increase in Cu (up to 25 wt.%) did not affect cell proliferation and differentiation^33^.

The addition of Cu has either been reported to improve the corrosion resistance of Ti alloys, for Cu < 3 wt.%^26^, or accelerate the corrosion rate of titanium alloys for Cu > 7 wt.%^34^, where the difference is primarily the amount of phases within the microstructure and their distribution. A comparable behaviour will be expected for the ternary Ti–xCu–yMn alloys due to their homogeneous microstructure, Cu concentration lower than 7 wt.%, and homogenous distribution of the phases in the microstructure.

**Figure 7:**
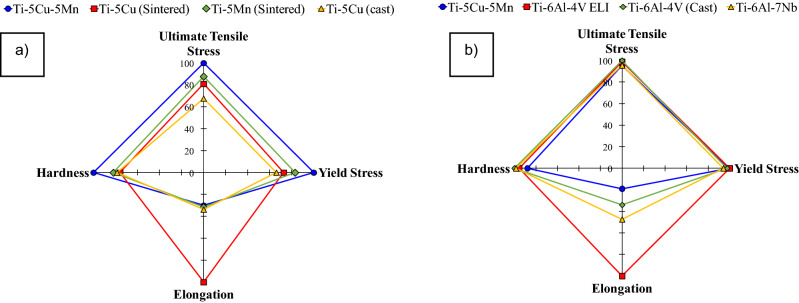
Four-factor radar chart comparing the performance of the β eutectoid bearing functionalised Ti alloys to other Mn- and Cu-bearing titanium alloys (**a**), and other conventional biomedical titanium alloys (**b**).

Ti-based alloys are greatly utilised as metallic structural biomaterials due to their excellent strength and biocompatibility; it is therefore of interest to compare the performance of the ternary Ti–xCu–yMn alloys to other Mn- and Cu-bearing titanium alloys (Fig. 7a) as well as other conventional titanium alloys (Fig. 7b). From Fig. 7a), the UTS and YS of the sintered Ti–5Cu–5Mn (UTS = 931 MPa and YS = 862 MPa) are higher compared to the sintered Ti–5Cu (UTS = 754 MPa and YS = 627 MPa)^21^ and the sintered Ti–5Mn (UTS = 817 MPa and YS = 716 MPa)^22^ materials. Moreover, sintered Ti–5Cu (9.5%) shows a higher elongation than Ti–5Cu–5Mn (2.8%), unlike Ti–5Mn (3%), whose elongation is comparable to that of the sintered Ti–5Cu–5Mn alloy. Following different preparation procedures, the mechanical properties of cast Ti–5Cu developed for dental applications^35^ show lower UTS (approximately 630 MPa) and YS (approximately 570 MPa) than the sintered Ti–5Cu–5Mn alloy with comparable elongation (3.2%).

Figure 7a also shows that the hardness of the sintered Ti–5Cu–5Mn (299 HV) alloy is higher than the sintered Ti–5Cu (227 HV), Ti–5Mn (245 HV), and cast Ti–5Cu (235 HV) alloys, which is beneficial to enhance the wear resistance of structural prostheses. With respect to alloys that are used extensively as orthopaedic implants in biomedicine, the sintered Ti–5Cu–5Mn alloy has comparable performance to cast Ti–6Al–4V (UTS = 976 MPa, YS = 847 MPa and elongation = 5.1%, ASTM F1108-88)^4^, mill-annealed Ti–6Al–4V ELI (UTS = 965 MPa, YS = 875 MPa and elongation = 15%, ASTM F136-84, F620-87)^36^, as well as to the Ti–6Al–7Nb alloy (UTS = 933 MPa, YS = 817 MPa and elongation = 7.1%, ASTM F1295-16)^37^. In terms of hardness, the sintered Ti5Cu-5Mn alloy (299 HV) is comparable with Ti–6Al–4V ELI (approximately 325 HV)^36^ and Ti–6Al–7Nb (300 HV) alloys^24^ but the cast Ti–6Al–4V has slightly higher hardness of 340 HV^38^.

Another critical feature that is important in this comparative analysis is the antibacterial property of the material, and specifically the antibacterial rate (Eq. 2 and Fig. 6). The sintered Ti–5Cu–5Mn alloy shows antibacterial rate of 97.6% against *E. coli*. which is comparable to current literature. Zhang et al.^39^ tested heat treated Ti–3Cu (T6) and Ti–4Cu (T6) alloys and found the antibacterial rate to be 90.3% and 92.6%. In another study^28^, the cast Ti–5Cu, as well as Ti–5Cu (T4), demonstrates antibacterial rates of 51% and about 67%, respectively. The heat-treated Ti–5Cu (T6) displayed a significant improvement in terms of antibacterial rate to ~ 94%. Finally, for sintered Ti–5Cu alloy, antibacterial rates of 99.0%—99.9%)^28,40^ were reported.

Overall, the β eutectoid bearing functionalised Ti alloys prove to be strong candidates for biomedical applications due to high mechanical and antibacterial properties. In addition, production of these alloys via powder metallurgy is highly beneficial and appealing due to the lower manufacturing time, energy consumption, and number of manufacturing steps used^41^ compared to more conventional metallurgical routes like casting and heat treatments. This opens up the possibility to manufacturing high-performance structural implants at lower cost made out of β eutectoid bearing functionalised Ti alloys.

Concluding, novel ternary Ti–xCu–yMn alloys were successfully synthesised via the simple and cost-effective powder metallurgy route entailing cold press and sinter. The progressive increment of Cu and Mn, elements biocompatible with the human body, does not significantly affect the processability of the material (as the final amount of porosity left is fairly constant) but drastically changes the phases composing the alloy. In particular, the equiaxed microstructure of titanium is transformed into a lamellar structure due to the stabilisation of the β-Ti phase (lamellar structure that is progressively refined by a high addition of alloying elements), and formation of Ti_2_Cu intermetallic particles occurs for sufficiently high amounts of Cu. Due to these microstructural changes, the strength and hardness of the β eutectoid bearing functionalised Ti alloys progressively increases but, conversely, the ductility eventually decreases. Consequently, the fracture mode of the ternary Ti–xCu–yMn alloys switches from ductile to transgranular and intergranular as a consequence of the reduction of the ability to tolerate damage. Regardless of the chemistry, the β eutectoid bearing functionalised Ti alloys show in vitro antibacterial activity against *E. coli*. Based on our findings, ternary Ti–xCu–yMn alloys produced via powder metallurgy offer the possibility of manufacturing cost-effective (as the cost is expected to be lower compared to other manufacturing processes) structurally sound Ti-based biomedical implants functionalised with strong antibacterial capability.

## Materials and methods

### Materials

Details of the raw materials for the creation of the β eutectoid bearing functionalised Ti alloys, which include high purity hydride-dehydride (HDH) Ti powder, high purity Cu powder, and high purity Mn powder, are reported in Table 1. The starting powders have different morphology (i.e. irregular for HDH Ti, dendritic for Cu, and angular for Mn) as consequence of the production method used to fabricate them. The irregular morphology of the HDH Ti powder, which constitutes the majority of the material, is the ideal shape to be processed via cold pressing so to obtain pressed products that can be safely be handled without premature failure.

**Table 1:** Characteristics of the starting powders for the production of the β eutectoid bearing functionalised Ti alloys.

Powder	Particle size (D_90_)	Mesh	Purity	Morphology
Ti (HDH)	75 μm	200	99.4%	Irregular
Cu	63 μm	230	99.7%	Dendritic
Mn	45 μm	325	99.0%	Angular

### Alloy design and samples preparation

In this study, a series of ternary Ti–xCu–yMn alloys was designed maintaining the addition ratio between Cu and Mn constant at 1 and progressively increasing the addition rate from 0.5 wt.% to 5 wt.%. The alloys were consequently labelled as: Ti–0.5Cu–0.5Mn, Ti–1Cu–1Mn, Ti–2Cu–2Mn, Ti–3.5Cu–3.5Mn, and Ti–5Cu–5Mn. The amount of each alloying element was determined and limited on the basis of the respective binary Ti–Cu and Ti–Mn phase diagrams, predicting the resulting phases^13^. CP Ti and Ti–5Cu samples, used respectively as negative and positive control during the quantification of the in vitro antibacterial activity, were also manufactured. The correct ratio of starting powders was weighted and blended at a speed of 45 Hz for 30 min at room temperature using a V-blender. The powder blends were subsequently poured into a 40 mm cylindrical steel die and cold pressed at room temperature applying a uniaxial pressure of 600 MPa using a 100-ton vertical hydraulic press. The pressed samples were then sintered under vacuum of 10^–3^ Pa at 1300 °C during 2 h of isothermal holding, using a heating rate of 10 °C/min and furnace cooling.

Samples with different geometry (for instance rectangular and dog-bone) were cut from the sintered Ti–xCu–yMn compacts using electric discharge machining (EDM). Specifically, rectangular samples were used for microstructural analysis via light optical microscopy and scanning electron microscopy (SEM), phases’ identification via X-ray diffraction (XRD) as well as for the plate count approach whilst dog-bone samples were used for tensile testing. It is worth mentioning that the surfaces of the samples were ground using Struers grit silicon carbide grinding papers to avoid any effect of the EDM surface finishing on the mechanical properties and to standardise the surface before testing the antibacterial response.

### Microstructural analysis and phases’ identification

Polishing of the samples for microstructural analysis was accomplished using a colloidal silica suspension. Moreover, the samples were etched using Kroll’s reagent (4% Hydrofluoric acid, 5% Nitric acid) prior to their microstructural analysis. For metallographic examination, an optical microscope equipped with a digital camera was used to obtain the morphology and distribution of residual porosity. A SEM coupled with an energy-dispersive spectrum (EDS) analyser, operated at 20 keV acceleration voltage, was used to investigate the microstructure, the fracture surface as well as to perform elemental analysis of the distribution of the chemical elements in the sintered samples to check the homogeneity of the composition. Phases’ identification via XRD was performed by means of a diffractometer with Cu K_α_ radiation. The 30–60°scanning range was analysed using a scan step size of 0.013° at 0.5 s per increment.

### Physical properties

The theoretical density of the alloys was calculated using the rule of mixture where the density of each element is multiplied by their weight percentage^22^. The density of the pressed samples (i.e. pre-sintering) was calculated by means of the weight and dimensions of the samples which were, respectively, measured using an analytical scale (4-decimal digits) and a digital calliper (2-decimal accuracy). The density of the sintered samples (i.e. post-sintering) was obtained by applying the Archimedes principle via the utilisation of the liquid displacement method according to ASTM B962. The amount of porosity was calculated as the difference between the value of the density and theoretical density of each alloy^41^.

### Mechanical behaviour

Tensile testing at room temperature of the sintered specimen was performed using a universal testing machine (load cell of 5 kN) using a cross-head test speed of 0.1 mm/min. A static axial strain gauge extensometer, with a gauge length of 10 mm, was utilised to obtain the strain value during tensile testing. YS of the β eutectoid bearing functionalised Ti alloys was calculated via the offset method as per ASTM E8-16. A minimum of three samples for each alloy was tested for consistency. Hardness tests were performed using the Rockwell A scale (HRA) making the indentations using a diamond indenter as per ASTM E18-19. For each sample, the test was repeated five times to ensure consistency and quantify the variation.

### In vitro antibacterial activity

#### Media, inoculum and lawn preparation

Luria Broth (LB) and agar plates were prepared aseptically according to the manufacturer’s recommendation (LIFE TECHNOLOGIES) and autoclaved for 15 min at 121 °C. A frozen *E. coli* DH5-α culture was streaked onto the surface of an LB agar plate using the four-quadrant method, inverted and grown overnight at 37 °C. A single bacterial colony was then selected to prepare the 10 mL LB inoculum to ensure genetically identical bacteria as well as eliminate any potential foreign bacterial contamination. A quality negative control was also prepared, LB broth with no bacteria inoculation, to confirm sterilisation of media and aseptic technique. The culture tubes were incubated overnight at 37 °C in a rotary shaker incubator at 200 rpm. Next, a lawn was prepared by adding 200 μL of the overnight bacterial culture onto the centre of the LB agar plate and spread evenly using a sterile glass spreader. The plate was then incubated for 30 min at room temperature to dry the surface of the agar and ensure inoculum absorption. Finally, the agar plates were inverted and incubated at 37 °C overnight.

#### Determination of bacterial concentration

Quantification of the concentration of the viable bacteria in the medium expressed in colony forming units (CFU) per millilitre was performed using the five-microlitre serial dilution spotting method^10^ in triplicative. Ten-fold serial dilutions (10^–2^ to 10^–8^) of overnight bacterial culture (OD_600_ = 0.8) were made using 1X PBS (Phosphate-Buffered Saline buffer), pH 7.4 and then 5.0 μL of diluted sample was spotted onto the LB agar plate with three drops per dilution. A 5.0 μL sample of undiluted bacteria (*E. coli* only) and a negative control (PBS only) were also included. The plates were then inverted in the incubator at 37 °C overnight from which the survived colonies were counted the next day. The acceptable range for total number of colonies is between 1–30 colonies. Equation 1 was used to obtain a CFU for each sample:

1 $$ {\varvec{CFU}}/{\varvec{mL}} = \frac{{\user2{Average\, number\, of\, colonies}}}{{\user2{Final\, plate\, dilution}}} = \frac{{\user2{Average\, number\, of\, colonies}}}{{\left( {\user2{Dilution\, factor\,*\,Inoculaiton}} \right)}}. $$

#### Plate count method

**Figure 8:**
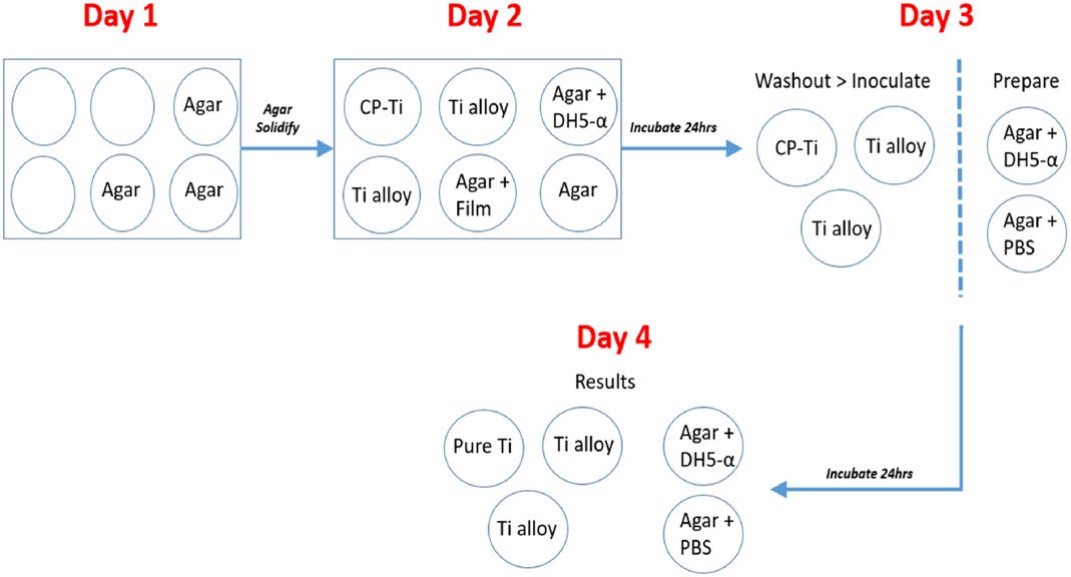
Detail of the experimental procedure used to prepare the specimens for the plate count assay.

The plate count method was conducted in vitro against *E. coli* under the guidance of Japan Industrial Standard JIS 2801: 2010 Antibacterial products—Test for antibacterial activity and efficacy^42^, as shown in Fig. 8, with minor modifications.

A six-well tissue culture plate containing bacterial cells (1–30 colonies) on nutrient agar (cell viability), agar only (to ensure no cross-contamination), and transparency film on top of the agar (confirm sterilisation of the film) was prepared. The remaining wells containing the test specimens with 30 μL of test inoculum was instilled onto each test specimen covered with 7 × 17 mm^2^ sterilised transparency film. The transparency film was sterilised before application by dipping into 70% isopropyl alcohol and then dried at 80 °C for 30 min in a sterile container with a slightly open cap. The aim for using the transparency film was to ensure a close homogenous surface contact of bacteria on the specimens as well as limiting bacterial evaporation and preventing leakage beyond the edges of the specimen. The six-well plate was then incubated overnight at 37 °C in a humidified chamber. Subsequently, the specimens were transferred to a sterile 10 mL screw cap polypropylene test tubes containing 2.2 mL PBS to extract the bacteria from the Ti specimen as well as the transparency film. Then, a 100 μL of the PBS washing solution was utilised to perform a serial dilution (up to 10^–6^) and plated onto nutrient and cultured in a humidified chamber overnight at 37 °C. After incubation, the CFU of the serially diluted plates was quantified between 30 to 300 per standard 90 mm diameter agar Petri dish^42,43^. The plates were imaged using a digital camera mounted on a repro stand for cell quantification purposes. From image analysis data, the maximal antibacterial reduction efficiency (*R*) was quantified via Eq. (2):

2 $$ {\varvec{R}} = 100\left( {1 - \frac{{{\varvec{CFU}}_{{{\varvec{sample}}}} }}{{{\varvec{CFU}}_{{{\varvec{reference}}}} }}} \right) $$

It is worth mentioning that three samples were tested for each material. The results, analysed statistically by a t-test, were considered statistically significant at *p < 0.05 and ^#^p < 0.01.

## Data Availability

All metadata pertaining to this work will be made available on request.
